# Genetic and Non-Genetic Contributions to Eosinophilic Granulomatosis with Polyangiitis: Current Knowledge and Future Perspectives

**DOI:** 10.3390/cimb46070446

**Published:** 2024-07-16

**Authors:** Mirko Treccani, Laura Veschetti, Cristina Patuzzo, Giovanni Malerba, Augusto Vaglio, Davide Martorana

**Affiliations:** 1 GM Lab, Department of Surgery, Dentistry, Gynaecology and Paediatrics, University of Verona, 37134 Verona, Italy; mirko.treccani@univr.it; 2 Infections and Cystic Fibrosis Unit, Division of Immunology, Transplantation and Infectious Diseases, IRCCS San Raffaele Scientific Institute, 20132 Milano, Italy; veschetti.laura@hsr.it; 3 Vita-Salute San Raffaele University, 20132 Milano, Italy; 4 Department of Neurosciences, Biomedicine and Movement Sciences, University of Verona, 37134 Verona, Italy; cristina.patuzzo@univr.it; 5 Nephrology and Dialysis Unit, Meyer Children’s Hospital IRCCS, 50139 Florence, Italy; augusto.vaglio@unifi.it; 6 Department of Biomedical Experimental and Clinical Sciences “Mario Serio”, University of Florence, 50121 Florence, Italy; 7 Medical Genetics Unit, Department of Onco-Hematology, University Hospital of Parma, 43126 Parma, Italy; dmartorana@ao.pr.it; 8 CoreLab Unit, Research Center, University Hospital of Parma, 43126 Parma, Italy

**Keywords:** EGPA, genetics, ANCA, eosinophils, Churg–Strauss syndrome

## Abstract

In this work, we present a comprehensive overview of the genetic and non-genetic complexity of eosinophilic granulomatosis with polyangiitis (EGPA). EGPA is a rare complex systemic disease that occurs in people presenting with severe asthma and high eosinophilia. After briefly introducing EGPA and its relationship with the anti-neutrophil cytoplasmic autoantibodies (ANCA)-associated vasculitis (AAVs), we delve into the complexity of this disease. At first, the two main biological actors, ANCA and eosinophils, are presented. Biological and clinical phenotypes related to ANCA positivity or negativity are explained, as well as the role of eosinophils and their pathological subtypes, pointing out their intricate relations with EGPA. Then, the genetics of EGPA are described, providing an overview of the research effort to unravel them. Candidate gene studies have investigated biologically relevant candidate genes; the more recent genome-wide association studies and meta-analyses, able to analyze the whole genome, have confirmed previous associations and discovered novel risk loci; in the end, family-based studies have dissected the contribution of rare variants and the heritability of EGPA. Then, we briefly present the environmental contribution to EGPA, reporting seasonal events and pollutants as triggering factors. In the end, the latest omic research is discussed and the most recent epigenomic, transcriptomic and microbiome studies are presented, highlighting the current challenges, open questions and suggesting approaches to unraveling this complex disease.

## 1. Introduction

Eosinophilic granulomatosis with polyangiitis (EGPA), firstly described by Churg and Strauss and formerly known as Churg–Strauss syndrome, is a rare complex systemic disease [1,2]. Together with granulomatosis with polyangiitis (GPA) and microscopic polyangiitis (MPA), EGPA is considered one of the anti-neutrophil cytoplasmic autoantibody (ANCA)-associated vasculitides (AAVs), a group of diseases characterized by the inflammation of blood vessels, tissue damage, loss of tolerance to neutrophil primary granule proteins and the consequent development of autoantibodies—the so-called ANCAs [3]. EGPA fulfills the definition of rare disease (fewer than 1 in 2000 individuals in any WHO region), equally affecting both males and females with a median age of onset of about 55 years old and with an estimated prevalence ranging between 2.0 and 22.3 cases per million people [4,5]. 

**Figure 1:**
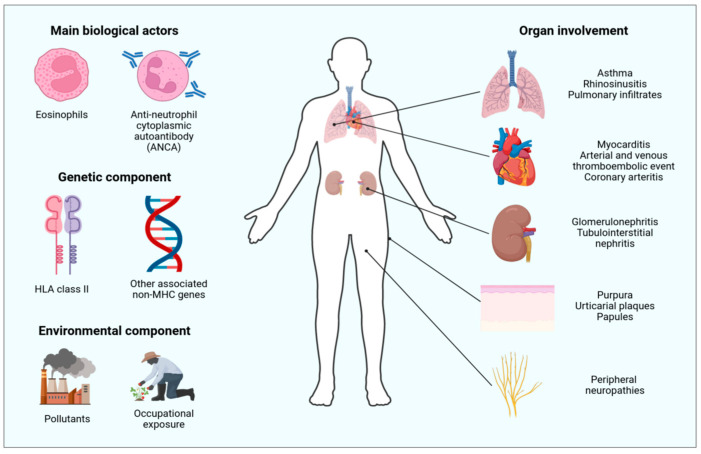
Graphical overview of the risk factors and organ involvement in eosinophilic granulomatosis with polyangiitis (EGPA). The figure illustrates the complex nature of EGPA, highlighting the most important biological factors, genetic and environmental influences, and the various affected organs described in this review.

EGPA is known to occur in people presenting with severe asthma and high eosinophilia, and it typically develops through three different phases. The initial phase is characterized by an allergic state, presenting asthma and atopy. The second phase represents the typical eosinophilic signature of EGPA, showing high eosinophil levels in different tissues. The third and last phase has more a vasculitis-like aspect, consisting of the necrotizing granulomatous inflammation of small-to-medium-sized vessels in various organs (such as the lungs, the skin, and the kidneys), which can be in different combinations [3,6]. The time span between asthma onset and EGPA diagnosis ranges between 5 to 9 years [7,8,9,10]. A general overview is provided in Figure 1.

As per their name, AAVs are characterized by the presence of anti-neutrophil cytoplasmic autoantibodies directed against two neutrophil primary granule proteins, proteinase 3 (PR3, also called myeloblastin) and myeloperoxidase (MPO), which are exposed during inflammation on the neutrophil cell surface [11]. AAVs vary significantly depending on the type and prevalence of ANCA: GPA predominantly presents with PR3-ANCA in 65–75% of cases, while MPA more frequently exhibits MPO-ANCA in 55–65% of cases. Both GPA and MPA have a low percentage of individuals who do not develop any autoantibodies (ANCA-negative).

Regarding EGPA, ANCA prevalence is estimated to be 30–40%, almost completely specific for MPO, thus causing MPO-ANCA-positive EGPA. Hence, the majority of EGPA-affected individuals are reported to be ANCA-negative [12]. Despite having a common core of high eosinophil levels and severe asthma, MPO-ANCA-positive patients show a more prominent autoimmune feature, being closer to a vasculitis; instead, ANCA-negative patients present dysfunctions in the mucosal barrier, leading to more eosinophilic infiltration and pointing at a more eosinophil-driven disease, hence showing a more allergic phenotype [13]. 

Eosinophils play a fundamental role in the onset and development of EGPA, and their presence represents a criterion for the definition of the disease, in accordance with the American College of Rheumatology [14,15]. Recent studies have suggested the existence of two different eosinophil-related facets of EGPA, named systemic and respiratory-limited phenotypes [16,17]. As reported by Matucci and colleagues, these two phenotypes have been identified using the blood eosinophil count as a disease biomarker. Both these subtypes showed the involvement of upper and lower airways together with the presence of asthma and chronic rhinosinusitis. However, the systemic phenotype presents the highest level of eosinophils and was reported to be associated with extra-pulmonary disease, involving other organ systems, such as the kidneys and the nervous system; instead, the respiratory-limited phenotype shows a lower level of blood eosinophils and the almost exclusive involvement of the lungs, leading to the development of eosinophilic alveolitis.

An overview of the EGPA subsets is reported in Table 1.

Currently, the determinants of EGPA are unknown. In the last decade, a growing number of studies have identified multiple genetic and environmental factors that may contribute to an increased risk of development of this disease, either in terms of genetic and environmental predispositions or as a trigger of immune dysregulation [11,18,19].

In this review, we delve into the genetics of EGPA. Starting from a general overview, we provide a comprehensive description of the research efforts, spanning from candidate gene studies to genome-wide association studies (GWASs), including familial cases. Additionally, we briefly discuss the environmental triggers of EGPA, offering glimpses into the non-genetic components of this disease. Finally, we summarize the latest omic findings, offering new insights and perspectives on future approaches to understand the genetic causes of EGPA.

## 2. The Genetics of EGPA: From Candidate-Gene to Whole-Genome Association Analyses

To date, two main genomic approaches have been used to investigate the genetic factors underlying EGPA and AAVs. At first, candidate-gene association studies were applied to investigate loci that were likely to be associated with these diseases, according to knowledge-based hypotheses. Then, with the advent of next-generation sequencing technologies, GWASs took over, allowing for the scanning of the entire genome to identify associated signals [20,21,22].

**Figure 2:**
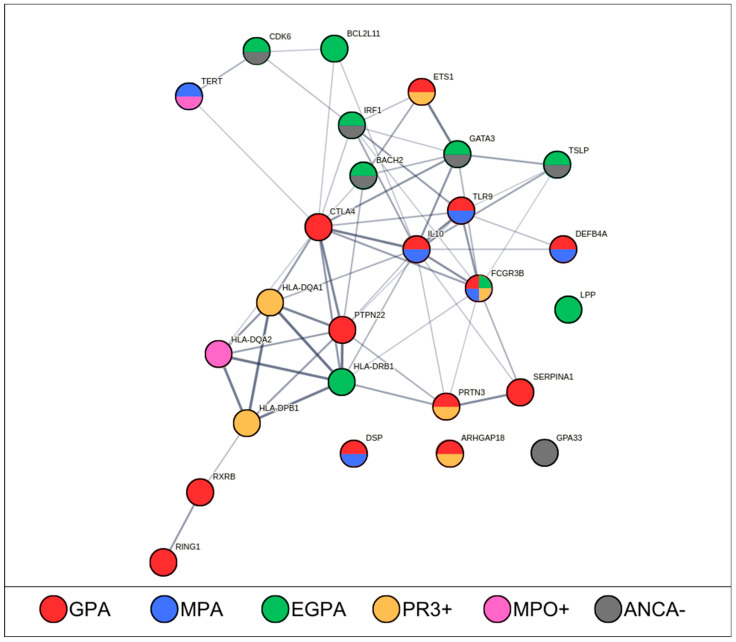
Network of the genes associated with AAVs. The figure shows a STRING [23] network representing the connection between the reported susceptibility genes. The displayed genes were reported at either suggestive (*p* < 5 × 10^−5^) or genome-wide (*p* < 5 × 10^−8^) significance in at least one genetic study. The majority of the genes belong to a common network whereas four genes (*DSP*, *ARHGAP18*, *GPA33*, *LPP*) do not show any connection. The different subsets of ANCA-associated vasculitis are encoded as follows: GPA = granulomatosis with polyangiitis (red); MPA = microscopic polyangiitis (blue); EGPA = eosinophilic granulomatosis with polyangiitis (green); PR3+ = PR3-ANCA-positive (yellow); MPO+ = MPO-ANCA-positive (magenta); ANCA- = ANCA-negative (gray).

These studies pointed out different candidate genes distributed along the genome, with particular regard but not limited to the major histocompatibility complex (MHC) region. Among the three AAVs, GPA is the most studied, and together with MPA, shows the clearest evidence of a genetic contribution and predisposition; instead, the genetics of EGPA remain largely unknown, mostly due to its rarity and complexity. An overview of all the genetic associations with all the three AAVs is provided in Figure 2.

Initially, candidate-gene studies were employed to dissect the genetic components of AAVs. Several genes were individually investigated on the basis of their biological functions, mostly related to inflammation and immunity.

One of the first candidate-gene studies focused on protein tyrosine phosphatase non-receptor type 22 (*PTPN22*), a gene which is involved in T-cell receptor signaling that showed associations in families affected by multiple autoimmune disorders [24]. Jagiello and colleagues identified an increased frequency in GPA-affected individuals compared to controls. This association was consequently confirmed by a second study [25], where cytotoxic T-lymphocyte-associated antigen 4 (*CTLA4*) was found to be associated with AAV, harboring susceptibility loci together with *PTPN22*; both these genes participate in T-cell signaling and regulation. Further immunoregulatory genes were investigated and were found to be associated with AAV syndromes. The ETS proto-oncogene 1 (*ETS1*), which is involved in the development of regulatory T cells, was found to be associated with both GPA and PR3-ANCA-positive AAV in the Japanese population [26]. Telomerase reverse transcriptase (*TERT*), which is involved in leukocyte telomere length and apoptosis, and desmoplakin (*DSP*), involved in immune response and carcinogenesis, showed increased-risk allele frequency and thus were reported to be associated with MPA and MPO-ANCA-positive AAV [27].

Other studies identified associations by not just investigating single nucleotide polymorphisms (SNPs) but focusing on copy number variations (CNVs). Defensin beta 4 (*DEFB4*) was reported to be associated with AAV in a Chinese cohort, showing an average increase in the genomic copy number compared to controls [28]. CNVs were also identified in Fc gamma receptor 3B (*FCGR3B*): a strong significant association was identified in both GPA and MPA, where individuals carrying a low copy number showed an increased risk of developing the diseases [29]. A similar trend was observed also in EGPA, where a low copy number was shown to predispose to EGPA [30] and was subsequently confirmed to increase the relapse risk [31].

Given the well-known role of the MHC region in immunity and antigen presentation, the first genetic studies investigated the impact of this region and its human leukocyte antigen (HLA) complex in AAVs [32,33,34], trying to assess the genetic contribution of the HLA genes to AAV susceptibility. Associations with HLA class II were identified in both EGPA and GPA: Vaglio and colleagues [35] found variants in *HLA-DRB1* and *HLA-DRB4* genes to be associated with EGPA, with increased frequencies compared to controls, and Heckmann and colleagues [36] identified that variants in the *HLA-DPB1* gene and the ring finger protein 1 (*RING1*)—a gene in close proximity to the HLA class II region—were associated with GPA.

Moreover, genes associated with AAV resulted from meta-analyses, which empowered signals with low significance or those unseen in single studies. Jung and colleagues [37] dissected the association between interleukin 10 (*IL10*) and vasculitis: the comparison of 21 studies led to the identification of SNPs that increased the susceptibility risk of AAV and specifically GPA. Consequently, Rahmattulla and colleagues [38] performed a genomic meta-analysis, investigating several genetic variants across multiple genes from 62 studies. This approach resulted in the identification of 33 variants associated with GPA and MPA that were located in or in close to candidate genes, such as *PTPN22*, *CTLA4*, *RING1*, and the HLA-DP locus, or were carried by previously not-significant genes taking part in either immune or inflammatory responses, such as the retinoid X receptor beta (*RXRB*), serpin family A member 1 (*SERPINA1*), toll-like receptor 9 (*TLR9*), and the HLA-DQ locus.

With the advent of new large-scale genotyping technologies, researchers started investigating variants alongside the whole genome and virtually covering all the human genes, shifting from candidate-gene to genome-wide approaches, increasingly combined with and empowered by genotype imputation [22]. To date, four GWASs have been performed on AAV syndromes, all investigating individuals of European descent—two focus on both GPA and MPA, one focuses on GPA only, and the most recent focuses exclusively on EGPA [11,18,39,40]. These GWASs confirmed some associations previously reported in candidate-gene studies, suggesting novel risk loci and allowing us to fine-map causative variants. 

The first GWAS ever performed on ANCA-associated vasculitis comprised a combined cohort of GPA- and MPA-affected individuals, divided into 1233 and 1454 cases and 5884 and 1666 controls in the discovery and replication cohorts, respectively [11]. In this study, Lyons and colleagues found associations in both the MHC and the non-MHC genomic regions. The GWAS confirmed the association (*p* < 5 × 10^−8^) of the HLA-DP locus, which was previously identified by Heckmann and colleagues with a gene-candidate approach [36]. Moreover, this study showed the association of two more genome-wide significant signals, one from the HLA-DQ locus, as previously identified by Rahmattulla and colleagues [38], and one from *SERPINA1*, encoding for alpha-1-antitrypsin, which is the main inhibitor of PR3. These genes turned out to be significant in AAV-affected patients as well as in GPA and PR3-ANCA-positive individuals. Moreover, genes at suggestive significance (*p* < 5 × 10^−5^) were also identified, confirming the association of *CTLA4*, as previously reported [25], and providing hints about novel associations, such as proteinase 3 (*PRTN3*), coding for PR3, and the Rho GTPase-activating protein 18 (*ARHGAP18*), involved in cell signaling.

A second GWAS comprising both GPA- and MPA-affected individuals was performed by Merkel and colleagues [39]. They analyzed 1986 AAV-affected individuals against 4273 healthy controls, focusing on the identification of functional and expression polymorphisms. This GWAS confirmed several genes that were previously reported to be associated at any significance level, such as the HLA-DP and the HLA-DQ loci in the MHC region and *SERPINA1*, *PTPN22* and *PRTN3* in the non-MHC region. Moreover, the study fine-mapped specific alleles of two HLA loci associated with AAV: HLA-DPA1 and *HLA-DPB1* of the HLA-DP locus, and *HLA-DQA1*, *HLA-DQA2* and *HLA-DQB1* of the HLA-DQ locus.

A third GWAS aimed at dissecting the genetic basis of GPA, investigating a cohort of about 492 cases and 1506 controls and replicating the results on a cohort of comparable size (528 cases and 1228 controls) [40]. Xie and colleagues identified 32 genome-wide significant associated signals, which were almost completely located in the MHC region. Both the alpha and beta chains of HLA-DP gene were found to be associated with GPA, as well as genes in close proximity to them, such as *RING1* and *RXRB*.

The fourth and last GWAS on AAV aimed to dissect the genetics of EGPA [18]. To date, this is the only GWAS focusing on this disease; due to its rarity, the recruitment of large cohorts of EGPA-affected individuals is difficult, making statistical analyses challenging. Lyons and colleagues enrolled 676 EGPA cases and 6809 controls, gaining insights not only into EGPA but also into MPO-ANCA-positive and ANCA-negative EGPA. They analyzed more than 9 million genetic variants, which resulted in three genome-wide significant associations with EGPA; the most significant signal was in the HLA-DQ locus, while the other two signals were in close proximity to gene regions—one on chromosome 2 within the BCL2 like 11 (*BCL2L11*) and the MIR4435-2 host gene (*MIR4435-2HG*), and on chromosome 5 near the thymic stromal lymphopoietin (*TSLP*) gene. Despite the difficulties in mapping causal variants lying outside the gene, Lyons and colleagues were able to provide biological meanings to the associations of *BCL2L11*/*MIR4435-2HG* and *TSLP* with EGPA. *BCL2L11* encodes for BCL2 interacting mediator of cell death (BIM) proteins, involved in apoptosis and immunity [41]; in addition, *MIR4435-2HG* encodes for a long non-coding RNA that regulates for BIM transcription and is involved in eosinophil apoptosis [42]. *TSLP* is instead involved in inflammation and drives eosinophilia; it has already been reported to be associated with asthma, eosinophils and allergic traits [43,44]. Moreover, some variants were also observed at suggestive significance and were consequently fine-mapped to identify the respective candidate genes: on chromosome 1 on the glycoprotein A33 (*GPA33*), responsible for barrier function in the lungs and in the intestine [45]; on chromosome 5 close to the Interferon regulatory factor 1 (*IRF1*) locus, involved in both innate and acquired immunity, and to the interleukin 5 (*IL5*) locus, involved in inflammation and in the growth and differentiation of eosinophils; on chromosome 6 on the BTB domain and CNC homolog 2 (*BACH2*), which plays a key role in regulating the maturation and differentiation of B and T cells; on chromosome 7 on the LIM domain-containing preferred translocation partner in lipoma (*LPP*), already reported to be associated with asthma and allergies [46,47], and with the cyclin-dependent kinase 6 (*CDK6*), involved in cell cycle regulation; in an intergenic region on chromosome 10 close to GATA binding protein 3 (*GATA3*), which is an important transcription factor expressed by several immune cells [48].

Lyons and colleagues focused not only on EGPA but also on its ANCA subsets, analyzing MPO-ANCA-positive and ANCA-negative EGPA. The clinical differences between the ANCA phenotypes were reflected in their genetics: on the one hand, MPO-ANCA-positive EGPA was strongly associated with the HLA-DQ locus; on the other hand, ANCA-negative EGPA was found to be associated with *BACH2*, *CDK6*, *GATA3*, *GPA33* and the *IRF1*/*IL5* locus. Thus, the associations with *BCL2L11*, *TSLP*, and *LPP* we found to be independent from the ANCA status. Hence, the genetic association resembled the specific ANCA features, proposing a more autoimmune profile in MPO-ANCA-positive EGPA, almost completely driven by the HLA complex, compared to a more eosinophilic and mucosal signature in ANCA-negative EGPA, characterized by the impact of non-MHC genes.

A complete overview of all the genetic associations with EGPA is provided in Table 2; an extended version of the table is available in Table S1.

Despite the number of genetic associations identified for EGPA, heritability and genetic predisposition remain largely unexplained, possibly due to the small effect as well as the rarity of the associated variants on the overall disease risk [49]. To answer these unsolved questions, family-based studies are needed. Following inheritance patterns and allele segregation, related people are more likely to be enriched for rare variants and thus could help in better explaining the genetic predisposition to complex diseases [50,51,52]. To date, only one family-based genetic study has been performed on EGPA, mostly due to the rarity of this disease and thus of familial cases. David and colleagues [53] took advantage of a well-characterized family that underwent whole-exome sequencing to identify causative genetic variants. The family presented four individuals, comprising a father and a son affected by EGPA and a mother and a daughter not presenting EGPA or any allergic symptoms. The study focused on variants that were transmitted following an autosomal dominant pattern and were likely to cause a loss of function or to have a pathogenic effect. A total of 65 rare variants, present only in the affected individuals, were identified; among them, only three were confirmed by both in silico predictions and in vitro experiments. These variants belonged to eosinophil peroxidase (*EPX*), the lipocalin 2 (*LCN2*), and Fms-related receptor tyrosine kinase 3 (*FLT3*). These genes were further investigated in 119 sporadic affected individuals, resulting in the identification of 24 different variants in *EPX* among 31 individuals. Thus, David and colleagues proposed *EPX* as a putative novel candidate gene, given its role in eosinophils homeostasis and its previous association with eosinophilic diseases [54,55,56].

At the current state of knowledge, EGPA is deemed to have a polygenic nature. Due to its rarity, it is likely that additional genetic factors still need to be identified, in order to explain its pathogenesis as well as to identify novel therapeutic targets.

## 3. Environmental Determinants of AAV and EGPA

As for other complex diseases, EGPA and in general AAVs have shown both genetic and environmental components, which are involved in their onset and development. To address the non-genetic causes, different studies have been carried out, mostly focusing on, but not limited to, GPA [57]. 

Seasonal variations in AAVs were investigated to assess a possible correlation between their onset and seasonal events, such as infectious diseases. Some studies identified a direct correlation between GPA and seasonal infections, with particular regards to the winter months, proposing the infectious event as one trigger for AAV. However, other studies identified this correlation in the summer months, proposing an allergic mechanism [58,59,60,61,62]. Thus, the correlation between AAV and seasonal events is still largely debated and needs to be further clarified.

In addition to seasonal events, the role of infections as a trigger for autoimmune diseases represents a research question in AAVs too. Infections from Epstein–Barr virus have been proposed as possible causative factors, particularly for MPO-ANCA-positive AAVs and EGPA [63,64]. Moreover, few case studies have reported the development of ANCA after severe acute respiratory syndrome coronavirus 2 (SARS-CoV-2) infection, thus suggesting SARS-CoV-2 infection as a trigger for autoimmunity, leading to the onset of AAVs [65,66,67,68,69]. Triggering infections have been identified also in bacteria, specifically in *Staphylococcus aureus* [70,71,72]. The presence of *S. aureus* has been associated with AAVs: some studies have reported that the colonization of the respiratory tract increases the susceptibility to GPA as well as the risk of relapses, probably due to the activation of the immune system as a response to the infection [70].

Pollutants have also been reported as potential determinants of AAVs [57]. A small number of studies have investigated different environmental compounds that may lead to the onset of these syndromes. Among them, Beaudreuil and colleagues [73] and Rihova and colleagues [74] identified a correlation with dust exposure, with particular regards to silica, which was subsequently confirmed in a meta-analysis by Scott and colleagues [75]. Other environmental risk factors have been identified in both rural and urban areas, despite the lack of strong evidence—in the former, this was due to the exposure to fertilizer and pesticides, and in the latter, this was due to the presence of high carbon monoxide levels and pollution [76,77].

Similar to its genetics, the environmental factors increasing the risk for AAVs are not fully understood and not yet completely identified. Hence, AAVs and EGPA etiology remain largely unclear. Given the nature of EGPA, environmental factors cannot be excluded from the triggering causes of this disease. While seasonal peaks and cyclical occurrences can be currently excluded, infections and exposures must be definitely taken into consideration [78]. Further research should focus on the role and impact of environmental triggers as well as their interaction with the genetic profile of affected people, to gain novel knowledge of the disease mechanisms.

## 4. Latest Research on EGPA

At the current state of knowledge, EGPA displays features of both vasculitis and eosinophilic disease. The advent of next-generation sequencing technologies and the rise of different omic approaches has made novel opportunities to dissect this disease available. Genomic association analyses were the first approaches put in place; however, epigenomic, transcriptomic and microbiome studies have been carried out in recent years to try to differentiate AAV syndromes. 

Two epigenetic studies have investigated the genes encoding for PR3-ANCA and MPO-ANCA, showing expression changes of *PRTN3* and *MPO* genes [79,80]. They focused on differentially expressed genes in AAV-affected individuals compared to healthy individuals that were correlated to *MPO* and *PRTN3* expression. Ciavatta and colleagues [79] identified an increased DNA methylation in ANCA patients, causing gene silencing and the dysregulated expression of *MPO* and *PRTN3*. Similarly, Yang and colleagues [80] identified a different histone modification pattern when comparing AAV patients and healthy controls, suggesting the involvement of epigenetic changes in AAV pathogenesis. 

A transcriptomic study focused on AAV to identify a specific transcriptional signature able to predict its onset [81]. McKinney and colleagues recruited 95 AAV-affected individuals and investigated the transcriptional profile of CD8 T cells. They identified differentially expressed genes linked to T-cell exhaustion and loss of function that were involved in the interleukin-7 receptor (*IL7R*) pathway and the T-cell receptor (*TCR*) signaling. Thus, they proposed CD8 T cells as possible prognostic biomarkers for AAVs.

More recently, Niccolai and colleagues [82] investigated the human gut microbiome in 29 EGPA-affected individuals. The analyses did not show any significant difference in the gut microbiome profiles of patients and controls. However, they showed an increase in bacterial species belonging to the *Enterobacteriaceae*, *Lactobacillaceae*, and *Streptococcaceae* families, which is likely to trigger intestinal inflammatory and immune responses in EGPA patients.

## 5. Conclusions and Future Perspectives

At the present time, EGPA largely remains an enigmatic disease. Despite technological and methodological advancements, which have allowed us to investigate and unravel the complexity of different human diseases and traits, the genetic and environmental factors triggering EGPA as well as its pathophysiological dynamics are poorly understood.

The environmental factors remain largely unexplained. Currently, only some evidence is available, but a consistent gap in the identification of all the possible triggering sources exists. Dissecting the non-genetic component of EGPA might help in our understanding of disease susceptibility. For example, the identification of the pathogens whose infections are likely to trigger AAVs could partially elucidate pathological mechanisms and related pathways. This might benefit from the investigation of the interaction between internal (i.e., genetics) and external (i.e., environmental) triggers, which will partially explain the complexity of this disease.

From the genetic perspective, multiple studies have shown the polygenic nature of EGPA. The GWAS on EGPA by Lyons and colleagues [18] proved that this disease is not only limited to the HLA locus, though representing the most significant signal, but also involves different genes outside the MHC genomic region. Hence, the validation of previously reported associations turns out to be an urgent need. The current genetic knowledge of EGPA can largely benefit from a second GWAS, and a subsequent meta-analysis of the resulting signals could confirm the true associations and clearly identify driver genes. Moreover, this will help to delineate the different genetic profile of MPO-ANCA-positive EGPA, which seems to be mostly related to the HLA contribution, and ANCA-negative EGPA, which seems impacted by non-MHC genes. However, the rarity of this disease and the consequent small number of affected individuals might make its realization challenging. Thus, despite not having a statistically powerful sample size, smaller studies can be of help in several ways. As suggested by David and colleagues [53], the analysis of families with EGPA-affected individuals will assist in the identification of rare variants. Although these variants are generally undetected in GWASs, tailored studies should be designed and performed, since they can explain a small fraction of the genetics of EGPA and thus provide an estimation of its heritability. Moreover, investigating families will prove the existence of a familial form of EGPA and eventually propose candidate genes that are likely to be associated with the familial form rather than with a sporadic form, as investigated by GWASs. However, given the polygenicity of EGPA, if a familial form of this disease exists, it is likely to have a genetic core shared with the sporadic form. 

Going beyond classical genetics and genomics, different omic approaches can provide novel insights to address the current open questions in the pathophysiology of EGPA. As for the other AAVs (GPA and MPA), epigenomics and transcriptomics offer novel perspectives and are helping in dissecting these diseases. Despite the recent advancements in genomics, epigenomics, and transcriptomics, these approaches have not been yet applied in investigating EGPA. Additionally, some basic research questions remain unsolved. The identification of all the genes associated with EGPA and harboring disease-causing variants is crucial. In addition to association studies and the consequent fine-mapping of pathogenic variants, the investigation of non-coding and regulatory regions might increase our understanding of this disease, shifting the focus from driver genes to their regulatory counterparts [83]. For example, the exploration of differentially expressed genes in EGPA-affected individuals compared to healthy controls could improve our understanding of the regulatory mechanisms taking place. Furthermore, the use of single-cell RNA sequencing [84,85] could help us to dissect the composition of the main biological actors of EGPA: ANCA and eosinophils. On the one hand, this could unravel the relationship between ANCA and EGPA; on the other hand, it could elucidate and validate eosinophils as novel clinical biomarkers.

Research carried out on similar inflammatory and autoimmune diseases can provide useful information on EGPA. The current knowledge of the other two AAVs (GPA and MPA), as well as on other eosinophilic diseases, such as asthma, allergies and hypereosinophilic syndrome, can be beneficial to advance our understanding of EGPA. For example, the investigation of genes belonging to the same pathway as well as those involved in similar functions across multiple diseases could help in dissecting the mechanisms triggering EGPA as well as suggesting novel targets. Moreover, the identification of these candidates could pave the way to drug repurposing [86]. The usage of drugs that have already been tested for similar diseases will dramatically decrease the cost related to drug discovery and development as well as confirm the identified targets, as proposed for systemic lupus erythematosus and AAVs [87].

Overall, the identification of novel candidate genes as well as the validation of already reported associations will further confirm the polygenic nature of EGPA, not only limited to the MHC region. The associated genetic signals coming from different omic studies might shift towards genetic scores [88,89]. The development of polygenic scores will help in estimating the individual susceptibility to diseases or traits according to genetic profiles. To date, there are several technical issues related to the development of a polygenic risk score tailored for EGPA, such as an adequate sample size for model training, testing and validation [90]. However, the combination of the associated genetic and environmental factors will help not only in the estimation of the susceptibility to EGPA but also in the development of personalized treatments and therapies.

## Figures and Tables

**Table 1:** Differences among EGPA subtypes. The table reports the currently known differences of the two EGPA subtypes in term of percentage of affected individuals, clinical phenotype, biological pathways, and genetics. Reported genes have been associated with the MPO-ANCA-positive or ANCA-negative phenotypes. All the associated genes are reported in Table 2.

Features	MPO-ANCA-Positive	ANCA-Negative
Affected individuals	30–40%	60–70%
Phenotype	Vasculitis-like	Allergy-like
Biological pathways	Autoimmunity	Mucosal dysfunction
Biological actors	ANCA and neutrophils	Eosinophils
Associated genes	*HLA-DQA2*, *TERT*	*BACH2*, *CDK6*, *GATA3*, *GPA33*, *IRF1*/*IL5*

**Table 2:** List of the genes (carrying variants or closest to intergenic variants) reported to be associated at genome-wide or suggestive significance with EGPA or ANCA status (in green). Chrom = Chromosome; EGPA = Eosinophilic granulomatosis with polyangiitis; PR3+ = PR3-ANCA-positive; MPO+ = MPO-ANCA-positive; ANCA- = ANCA-negative.

Gene	Chrom	EGPA	PR3+	MPO+	ANCA-
*FCGR3B*	1				
*GPA33*	1				
*BCL2L11*	2				
*MIR4435-2HG*	2				
*LPP*	3				
*IRF1*	5				
*TERT*	5				
*TSLP*	5				
*ARHGAP18*	6				
*BACH2*	6				
*HLA-DPB1*	6				
*HLA-DQA1*	6				
*HLA-DQA2*	6				
*HLA-DRB1*	6				
*HLA-DRB4*	6				
*CDK6*	7				
*GATA3*	10				
*ETS1*	11				
*PRTN3*	19				

## Supplementary Materials

The following supporting information can be downloaded at: https://www.mdpi.com/article/10.3390/cimb46070446/s1, Table S1: Extended list of the genes reported to be associated with one or more ANCA-associated vasculitis or subtypes.
